# An effective method to generate controllable levels of ROS for the enhancement of HUVEC proliferation using a chlorin e6-immobilized PET film as a photo-functional biomaterial

**DOI:** 10.1093/rb/rbab005

**Published:** 2021-03-13

**Authors:** Seung Hee Hong, Min-Ah Koo, Mi Hee Lee, Gyeung Mi Seon, Ye Jin Park, HaKyeong Jeong, Dohyun Kim, Jong-Chul Park

**Affiliations:** 1 Cellbiocontrol Laboratory, Department of Medical Engineering; 2 Department of Medical Engineering, Graduate School of Medical Science, Brain Korea 21 Project; 3 Department of Medical Device Engineering and Management, Yonsei University, College of Medicine, Seoul 03722, Republic of Korea

**Keywords:** reactive oxygen species, chlorin e6, dopamine, covalent bond, HUVEC proliferation

## Abstract

Reactive oxygen species (ROS) are byproducts of cellular metabolism; they play a significant role as secondary messengers in cell signaling. In cells, high concentrations of ROS induce apoptosis, senescence, and contact inhibition, while low concentrations of ROS result in angiogenesis, proliferation, and cytoskeleton remodeling. Thus, controlling ROS generation is an important factor in cell biology. We designed a chlorin e6 (Ce6)-immobilized polyethylene terephthalate (PET) film (Ce6-PET) to produce extracellular ROS under red-light irradiation. The application of Ce6-PET films can regulate the generation of ROS by altering the intensity of light-emitting diode sources. We confirmed that the Ce6-PET film could effectively promote cell growth under irradiation at 500 μW/cm^2^ for 30 min in human umbilical vein endothelial cells. We also found that the Ce6-PET film is more efficient in generating ROS than a Ce6-incorporated polyurethane film under the same conditions. Ce6-PET fabrication shows promise for improving the localized delivery of extracellular ROS and regulating ROS formation through the optimization of irradiation intensity.

## Introduction

Photodynamic therapy (PDT) is a non-invasive treatment for cancer with minimal side effects. PDT has a relatively low toxic effect on the biological system and does not have a repeatability of cumulative toxicity. During PDT, a specific wavelength of light excites photosensitizers in the presence of molecular oxygen, and photosensitizers specifically localize in the target tissue [1–5]. PDT leads to the production of reactive oxygen species (ROS), which play an essential role as regulatory mediators in the signaling process of cells and tissues [6, 7]. *In vitro*, the effect of PDT is strongly related to the concentration of photosensitizers, which affects the quantity of ROS production. Photosensitizers could only produce a limited amount of ROS at low concentrations, whereas they could generate enough ROS to treat cancer at high concentrations. ROS confers cells advantages or disadvantages depending on the amount of ROS produced. A high amount of ROS results in apoptosis or necrosis through irreversible damage to biomolecules, such as DNA, protein, and RNA, whereas a low amount of ROS promotes cellular proliferation. Consequently, modulating the generation of ROS is vital to their role in cellular function [8–13].

Chlorin e6 (Ce6) is a photosensitizer synthesized from chlorophyll with high efficacy and minimal toxicity [14–20]. Ce6 has strong absorption in long-wavelength light, such as red light. The use of long wavelengths significantly improves the penetration depth compared with the use of short wavelengths [21]. For this reason, Ce6 allows deep lesion treatment. However, Ce6 is disadvantageous for medical use because of its low solubility, compared with other photosensitizers [22]. To circumvent this downside of Ce6, it is necessary to improve its solubility or develop an efficient Ce6-delivery system. There are several improved delivery systems, such as polymers, gold nanoparticles, silicon nanoparticles and liposomes [23–29].

Dopamine is widely used for surface modification of polymers, ceramics or metals [30, 31]. The application of dopamine on the surface involves various interactions between natural or synthetic materials and bioactive molecules [32–34]. Dopamine is easily oxidized to polydopamine. Polydopamine comprises uncyclized amine-containing moieties and cyclized indole type units, linked together via both covalent and non-covalent interactions [35]. Through these functional groups on polydopamine, biomolecules can be easily combined. The amino group of polydopamine could form an amide bond through 1-ethyl-3-3(3-dimethylaminopropyl) carbodiimide hydrochloride (EDC)/N-hydroxysuccinimide (NHS) chemistry as a covalent bond. Covalent bonding strengthens the linkage of photosensitizers to the support, reducing the problem of leakage or desorption. In particular, activation with carbodiimide could lead to the production of biomolecules with improved resistance to degradation.

In this study, we designed a Ce6-immobilized PET film (Ce6-PET) through covalent bonding. Ce6 was conjugated with the amino group of dopamine via EDC/NHS chemistry. We confirmed the possibility of using the Ce6-PET film to control the generation of ROS for specific lesions by adjusting the LED power. In addition, we demonstrated the effect of human umbilical vein endothelial cell (HUVEC) proliferation at optimum ROS levels through Ce6-PET film. Therefore, we compared the ROS generation efficiency of the Ce6-PET film and Ce6-incorporated polyurethane (Ce6-PU) films.

## Materials and methods

### Ce6-PET film preparation

**Figure 1.**
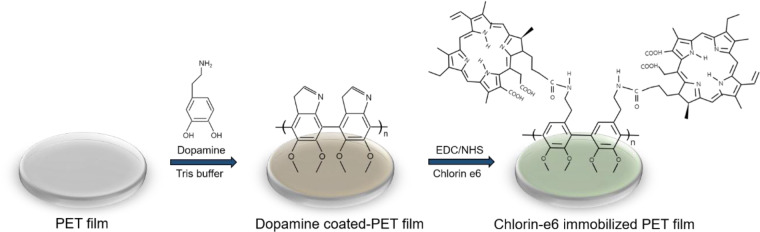
Schematic of the Ce6-PET fabrication method.

The polyethylene terephthalate (PET) film prepared was 1 cm^2^ in size, with a thickness of 0.1 mm (Goodfellow, Huntingdon, England). To prepare a polydopamine coating solution, dopamine hydrochloride (Sigma-Aldrich, St Louis, MO, USA) was dissolved at a concentration of 1 mg/ml in 10 mM pH 8.5 Tris-HCl buffer (Biosesang, Seongnam, Korea). The PET film was immersed in the dopamine solution for 18 h at room temperature. The dopamine-coated PET film was washed with distilled water (DW) five times and air-dried. The film was immersed in a solution containing Ce6 mixtures (Santa Cruz, CA, USA), EDC (Sigma-Aldrich) and NHS (Sigma-Aldrich) in DMSO with constant shaking for 24 h at room temperature (Fig. 1). The immobilized surfaces were sterilized in 70% ethanol (EtOH) for 30 min and rinsed with DW. The Ce6-PET film was wrapped with aluminum foil to avoid light.

### Light emitting diode power controller

A self-designed red light emitting diode (LED) system was used as a light source to control irradiation, as previously reported [36]. The emission peak of the red LED light at 660 nm corresponded to the maximum absorption of Ce6 in the visible light region. This system was also designed with filters between the LED source and the bottom to eliminate ultraviolet radiation. Samples were consistently irradiated with the LED underneath at a distance adjusted so as to give an irradiation spot of 4.5 cm. The LED’s output power was measured and adjusted to the desired setting power for each assay using an optical power meter (THORLAB, NJ, USA) prior to the experiment.

### Ce6-PET film characterization

The amount of dopamine coated on the PET film was determined using a micro-BCA protein assay kit (ThermoFisher, MA, USA). The BCA working agent was reacted with 1 cm^2^ of the dopamine-coated PET film sample for 2 h at 37°C inside an incubator. The reagent was detected at 562 nm using a microplate reader (Molecular Device, CA, USA). The coating morphology of dopamine on the PET film surface was observed by scanning electron microscopy (FE-SEM, Merlin, Carl Zeiss, Germany). To compare the ROS generation ability between different dopamine coating concentrations, a 1,3-diphenylisobenzofuran (DPBF; Sigma-Aldrich) degradation assay was conducted. The PET film was prepared in two different ways: two concentrations of dopamine coating (1 and 2 mg/ml) with the same concentration of Ce6-EDC-NHS were reacted. The sample was dipped in DPBF solution and was irradiated with 660 nm LED light at a power of 10, 20 and 30 mW/cm^2^ for 10 min. The DPBF solution was transferred to a cuvette and the UV-vis absorbance spectrum was recorded (Shimadzu, Kyoto, Japan).

A Fourier-transform infrared (FTIR) spectrometer was used to analyze the chemical structure of the PET film surface. The Ce6-PET film was placed in a holder, and the spectra were collected using an FTIR spectrometer (FT/IR 4600, JASCO, Tokyo, Japan). The spectrum was taken in the range of 4000–900 cm^−1^ in transmission mode, and the resolution was 4 cm^−1^.

To confirm ROS generation via the Ce6-PET film, DPBF, which is an active oxygen quencher, was detected. The average power of the controller was measured using an optical power meter. The irradiation power was 200, 500 μW, 1, 2 and 5 mW at 1 cm^2^ in each experiment. The Ce6-PET film was irradiated for 30 min in the dark. The optical density (OD) of the DPBF solution was determined by UV-vis absorbance spectroscopy (Shimadzu, Kyoto, Japan).

The amount of Ce6 immobilized on the dopamine-coated PET film was analyzed by fluorescence. The Ce6-PET film was dipped in 1 M NaOH for 3 days to dissolve the dopamine coating. The dissolved Ce6 was then quantified by measuring fluorescence at 405 nm (excitation) and 670 nm (emission) using a fluorescence microplate reader (Flexstation3, Molecular Device, Union City, CA, USA).

### Cell culture and incubation conditions

HUVECs were purchased from Lonza (Basel, Switzerland) and cultured in endothelial cell growth basal medium with supplements at 37°C in a 5% CO_2_ atmosphere in a humidified incubator. Culture media was replaced with fresh medium every 3–4 days. When cells reached 90% confluence in the flask, they were detached using 0.25% trypsin-EDTA and replaced. HUVECs below passage six were used for the experiment.

### HUVEC proliferation

HUVECs were seeded on Ce6-PET film at a density of 1 × 10^4^ cells/well. Cells were incubated overnight at 37°C, and then irradiated with red LED light for 30 min. After irradiation, cell viability was assessed using the 3-(4,5-dimethylthiazol-2-yl)-2,5-diphenyl-2-H-tetrazolium bromide (MTT) assay at 0, 1, 3 and 5 days, respectively. MTT reagent was mixed with EGM-2 and incubated in the dark for 4 h. MTT solution was absorbed into the cells, and DMSO was added to release purple formazan crystals by dissolving the cells. It was transferred to 96-well plates, and its absorbance was measured on a microplate reader at a wavelength of 570 nm (Molecular Device, CA, USA).

### Ce6-PU film fabrication

Ce6-incorporated PU films were prepared by a solvent casting method. The polymer solution was prepared by dissolving Elastollan pellets in tetrahydrofuran (THF; Junsei, Japan) at room temperature. The amount of incorporated Ce6 was determined based on the amount of Ce6 immobilized, which was evaluated from the Ce6-PET film. The Ce6-PU film was fabricated at four different Ce6 concentrations; the same amount on film immobilized by covalent bonding (1 × Ce6-PU), twice that of Ce6-PET (2 × Ce6-PU), five times more than Ce6-PET (5 × Ce6-PU), and ten times more than Ce6-PET (10 × Ce6-PU). The required amount of Ce6 was dissolved in DMSO and mixed with THF. When the Ce6 was fully dissolved, the Elastollan pellets were added and dissolved. The final polymer solution concentration was 10%. The polymer solution was air-dried for 3 days and punched into 1 cm^2^-sized films. The Ce6-PU film was sterilized by 70% EtOH for 30 min and washed five times with DW. The Ce6-PU film was stored at 4°C and wrapped with aluminum foil to avoid light.

### Ce6-PU film efflux test

Ce6 released from the Ce6-PU film during the sterilizing procedure was investigated through fluorescence. Four different concentrations of Ce6-incorporated PU films were used to determine the release pattern. During each step of the sterilizing procedure, 70% EtOH and DW were gathered, and DMSO was added to collect Ce6 in EtOH and DW.

To investigate the *in vitro* release of Ce6 from the PU film, the Ce6-PU film was dipped in PBS and incubated at 37°C under continuous shaking conditions. At specific time intervals within three days, DMSO was added to the collected PBS to collect the Ce6. Normalized peak fluorescence was measured over 3 days, and the cumulative release amount of Ce6 was plotted. The fluorescence of Ce6 was measured using a fluorescence microplate reader (Flexstation3, Molecular Device, Union City, CA, USA). The result was calculated from the standard calibration curve of Ce6.

In addition, to evaluate the influence of the released Ce6-PU on the media, HUVEC viability was measured. HUVECs were seeded at 2 × 10^4^ cells/cm^2^ in 48-well plates and incubated for 24 h. At the same time, Ce6-PU film was placed in media for 24 h at 37°C. After 24 h, the collected media were used to culture HUVECs for 2 h. After 2 h, the cells were irradiated with red LED light. Cell viability was measured by MTT 1 or 2 days after light irradiation.

### Comparison of ROS production

A decomposition study of DPBF was performed on Ce6-PET film and Ce6-PU film that generated ROS upon red LED irradiation. One milliliter of DMSO solution containing DPBF was introduced onto the Ce6-PET and Ce6-PU films in the dark. A red LED light source was used to irradiate the Ce6-PET and Ce6-PU films, and the LED power used was 200, 500 μW, 1, 2 and 5 mW at 1 cm^2^ in each experiment. The irradiation time was fixed at 30 min. The OD of the DPBF absorption peak at 411 nm was monitored by UV-vis spectroscopy (Shimadzu, Kyoto, Japan).

### Statistical analysis

All data are presented as means ± SDs. Statistical analyses were performed using SPSS 23.0 software. A comparison within the studied groups was performed using the paired t-test, while comparisons between groups were performed by one-way analysis of variance. A value of *P* < 0.05 was considered statistically significant.

## Results

### Characterization of immobilized Ce6 on PET film

**Figure 2.**
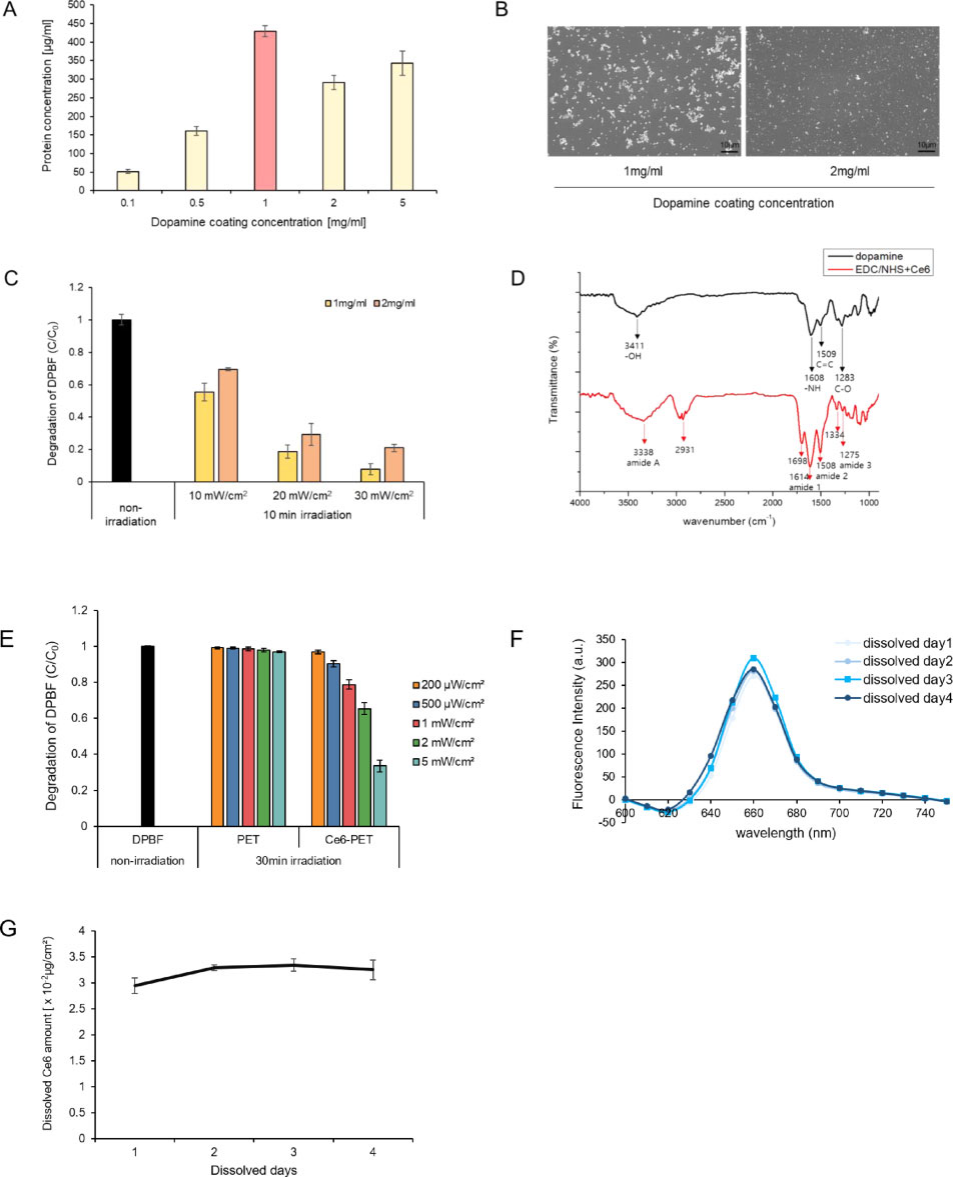
Characteristics of the Ce6-PET film. (**A**) Concentration of dopamine coated on the PET film was determined by the micro BCA assay. Varying concentrations were coated onto the PET film. Data are expressed as the mean ± SD. (**B**) SEM image of the dopamine-coated PET surface. Dopamine (1 mg/ml and 2 mg/ml) was coated onto the PET film. Scale bar = 10 µm. (**C**) DPBF degradation was compared with Ce6 immobilized on two types of dopamine-coated film following red-light irradiation at different intensities for 10 min. Data are expressed as the mean ± SD. (**D**) FTIR spectrum of dopamine-coated PET film and Ce6 immobilized through EDC/NHS-coupled PET film. The black line indicates the dopamine-coated PET film. The red line indicates the Ce6-PET film. (**E**) The ratio between initial OD and decomposition OD is shown as irradiation power. DPBF decomposition in the samples containing DPBF alone and DPBF with the Ce6-PET film following irradiation with different intensities for 30 min was compared. Data are expressed as the mean ± SD. (**F**) Fluorescence spectrum of Ce6-PET film dissolved days (excitation, 405 nm; emission, 600–750 nm). (**G**) Total amount of Ce6 dissolved in Ce6-PET was calculated using the Ce6 standard curve. Data are expressed as the mean ± SD.

In order to determine the optimal dopamine coating concentration, different concentrations of dopamine solution were prepared to immerse the PET film. A micro BCA assay was conducted to determine the amount of dopamine coated on the PET film [37]. The film that was immersed in 1 mg/ml dopamine solution yielded the highest amount of dopamine coating compared with the other films with different concentrations of dopamine solution (Fig. 2A).

The SEM images show the quantity of dopamine layered on the PET film. Through the SEM image, we observed the changes in the surface morphology of the dopamine-coated film, including its size and shape, under the two different concentrations of dopamine. On the 1 mg/ml dopamine-coated PET film, dopamine was polymerized and aggregated, but only a small amount of dopamine was enveloped on the 2 mg/ml dopamine-coated PET film (Fig. 2B).

To compare the Ce6 immobilizing efficiency of dopamine among different concentrations, 1 and 2 mg/ml dopamine-coated films were immersed in the same concentration of Ce6-EDC-NHS solution to immobilize Ce6 through covalent bonding. A DPBF decomposition assay was conducted to compare the ROS generation indirectly. DPBF degradation indirectly assumes ROS generation in the external cellular environment. Production of ROS by Ce6 could be determined by monitoring the photo-oxidation of DPBF upon light exposure. As an ROS quencher, DPBF undergoes a 1,4-cycloaddition reaction with ROS to form an endoperoxide that decomposes into the irreversible product 1,2-dibenzoylbenzene [38]. C_0_ represents the DPBF solution without light irradiation. C/C_0_ represents the absorbance change ratio due to ROS generation. Two types of films were prepared, and each film was dipped in DPBF solution. Each film was subjected to red-light irradiation at intensities of 10, 20 and 30 mW/cm^2^ for 10 min. As shown in Fig. 2C, under the same LED irradiation power and time, Ce6 immobilized on the 1 mg/ml dopamine-coated film showed more DPBF degradation than that in case of the 2 mg/ml dopamine-coated film. The 1 mg/ml dopamine solution coating on the PET film allowed more functional groups to undergo EDC/NHS coupling. Thus, the 1 mg/ml dopamine-coated film could generate ROS more efficiently at higher concentrations of immobilized Ce6. Through the micro BCA assay, SEM image, and DPBF degradation results, we selected a 1 mg/ml dopamine-coated PET film because of the greater opportunities for creating a covalent bond with a functional group compared with other films at different concentrations.

FTIR spectroscopy was performed to understand the surface chemical structure of the Ce6-PET film as well as the binding interactions between the dopamine-coated PET film and the Ce6 immobilized by EDC/NHS coupling onto the dopamine-coated PET film. The spectrum of the dopamine-coated PET film showed well-defined peaks that appeared in the spectrum of the polydopamine. However, the peaks were slightly shifted and had different relative intensities without affecting the surface chemical structure of the film. The bands at 3411, 1608, 1509 and 1283 cm^−1^ correspond to the stretching vibrations of the –OH, N-H group, aromatic C = C stretching and C-O stretching in the polydopamine, respectively. The activated carboxyl groups on Ce6 were covalently coupled with the amine groups of dopamine by the EDC/NHS coupling reaction to prepare the Ce6-immobilized PET film. Absorption bands associated with the presence of amides were identified. The bands at 3338, 1614, 1508 and 1275 cm^−1^ correspond to N-H stretching coupled with hydrogen bonding (amide A), C = O stretching (amide 1), N-H band coupled with C = N stretch (amide 2) and N-H band (amide 3). The Ce6 spectrum band is also represented at 2931, 1698 and 1334 cm^−1^ (Fig. 2D). These results indicated that amide bonds and Ce6 were successfully introduced on the dopamine-coated PET film surface [39–45].

Ce6-PET film was prepared and DPBF degradation was conducted to determine the total ROS generation. In the presence of ROS, the yellow compound, DPBF, was oxidized to a colorless compound, 1,2-diphenylbenzene. DPBF can be decomposed by superoxide anion radicals, singlet oxygen, hydrogen peroxide and other ROS [46]. The difference in absorbance before and after exposure to light was taken as the measure of DPBF quenching [47]. ROS generation from Ce6-PET under the red LED was assessed by observing the power-dependent photodegradation of DPBF. The DPBF solution was irradiated by the red LED for 30 min at a power of 200, 500 μW/cm^2^, 1, 2 and 5 mW/cm^2^. As shown in the DPBF degradation assay, Ce6-PET film decomposed DPBF depending on the light power (Fig. 2E).

Dopamine-coated PET film was used to immobilize Ce6 using a covalent bond resulting from immersing the film in a Ce6-EDC-NHS mixture. To measure the amount of immobilized Ce6, the film was dipped in 1 M NaOH [48]. Dopamine was fully dissolved in 1 M NaOH for 3 days to analyze the amount of immobilized Ce6 on the PET film. After dopamine covered the PET film, further addition of NaOH was required to disassemble dopamine. Through NaOH, the amine group of dopamine was deprotonated and delaminated from the PET film [49]. Therefore, the extent of disassembled dopamine and dissolved Ce6 fluorescence could indicate the amount of Ce6 immobilized. Through the fluorescence spectrum, we confirmed that the dissolved film for 3 days showed the highest amount of immobilized Ce6 (Fig. 2F). Thus, the dopamine-coated PET film was fully dissolved in 3 days. To prepare the Ce6 standard curve, Ce6 was diluted ten-fold with NaOH and then fluorescence was measured. By using the Ce6-NaOH diluted standard curve, we assumed that 3.3 × 10 ^−2 ^μg of Ce6 was immobilized on 1 cm^2^ of PET film (Fig. 2G). This result was used to fabricate the PU film, which contained the same amount of Ce6.

### HUVEC proliferation through ROS generated from Ce6-PET film

**Figure 3.**
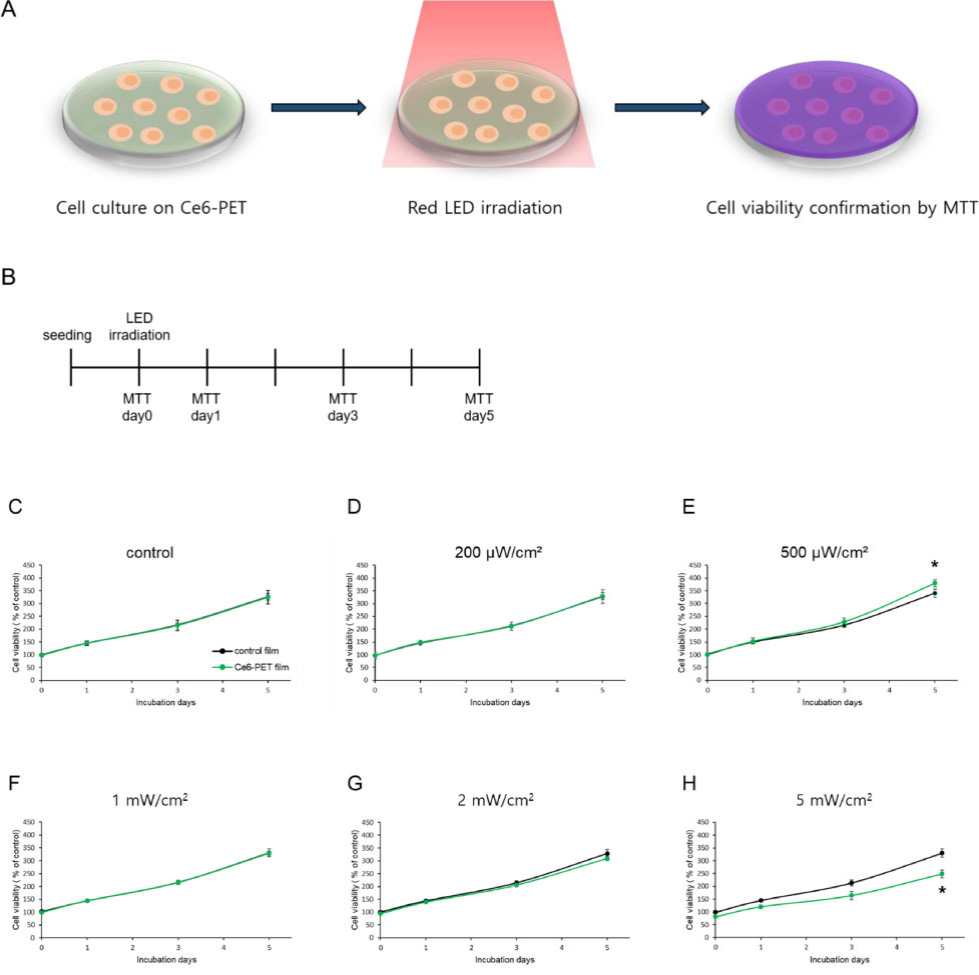
HUVEC Proliferation assessment on the Ce6-PET film following red LED irradiation. (**A**) Schematic of the experimental method. (**B**) Timeline of the experimental schedule. Cellular proliferation induced by ROS was measured by the MTT assay. The black line indicates the cell proliferation in the control film, and the green line indicates the cell proliferation on the Ce6-PET film. Cells were irradiated with different light power levels for 30 min: (**C**) without LED irradiation, (**D**) 200 μW/cm^2^, (**E**) 500 μW/cm^2^, (**F**) 1 mW/cm^2^, (G) 2 mW/cm^2^ and (H) 5 mW/cm^2^. Data are expressed as the mean ± SD, * *P* < 0.05 vs. control film.

ROS is an inevitable byproduct of metabolism. ROS have different effects on cells depending on its concentration. High levels of ROS are hazardous to cells and their contents. In contrast, a low level (physiological level) of ROS is essential to regulate cell functions and proliferation, acting as a second messenger in a cellular pathway. To confirm the difference in HUVEC growth between Ce6-PET film and dopamine-coated film (control film), Ce6-PET and control film was irradiated with varying power levels of red LED to produce controllable ROS in the extracellular environment. The Ce6-PET film managed ROS generation through precision control. Cellular proliferation by Ce6-PET film was evaluated by the MTT assay. HUVECs were cultured on Ce6-PET film and control film. HUVECs were irradiated with 660 nm of red light from the LED at varying power levels for 30 min. This experiment showed that light irradiation at a power of 500 µW/cm^2^ on HUVECs that were seeded on the Ce6-PET film significantly increased the cellular growth, compared with those seeded onto the bare film on day 5 (Fig. 3). Power levels less than 500 µW/cm^2^ did not affect HUVEC growth, while powers higher than 500 µW/cm^2^ caused cell growth inhibition. This result reveals that extracellular ROS could affect cellular signaling and induce cell growth. Thus, using Ce6-PET film with LED irradiation is a simple method to modulate the production of ROS, which could be used to control cellular behavior.

### Ce6 released from the Ce6-incorporated PU film

Here, we compared efficiencies between the entrapment method and the covalent bonding method. We fabricated Ce6-incorporated PU film for the entrapment method and Ce6-PET film for the covalent bonding method.

The photosensitizer released from the PU film caused potential side effects on local delivery for medical treatment [50, 51]. Therefore, it is important to control the release of photosensitizers. Ce6 release from the Ce6-PU film was evaluated. Four different concentrations of Ce6-PU film (1×, 2×, 5× and 10× multiple amounts corresponded to Ce6-PET film; 1× Ce6-PU; 3.3 × 10^−2 ^μg/cm^2^, 2× Ce6-PU; 6.6 × 10^−2 ^μg/cm^2^, 5× Ce6-PU; 16.5 × 10^−2 ^μg/cm^2^, 10× Ce6-PU; 33 × 10^−2 ^μg/cm^2^) were investigated in this study.

**Figure 4.**
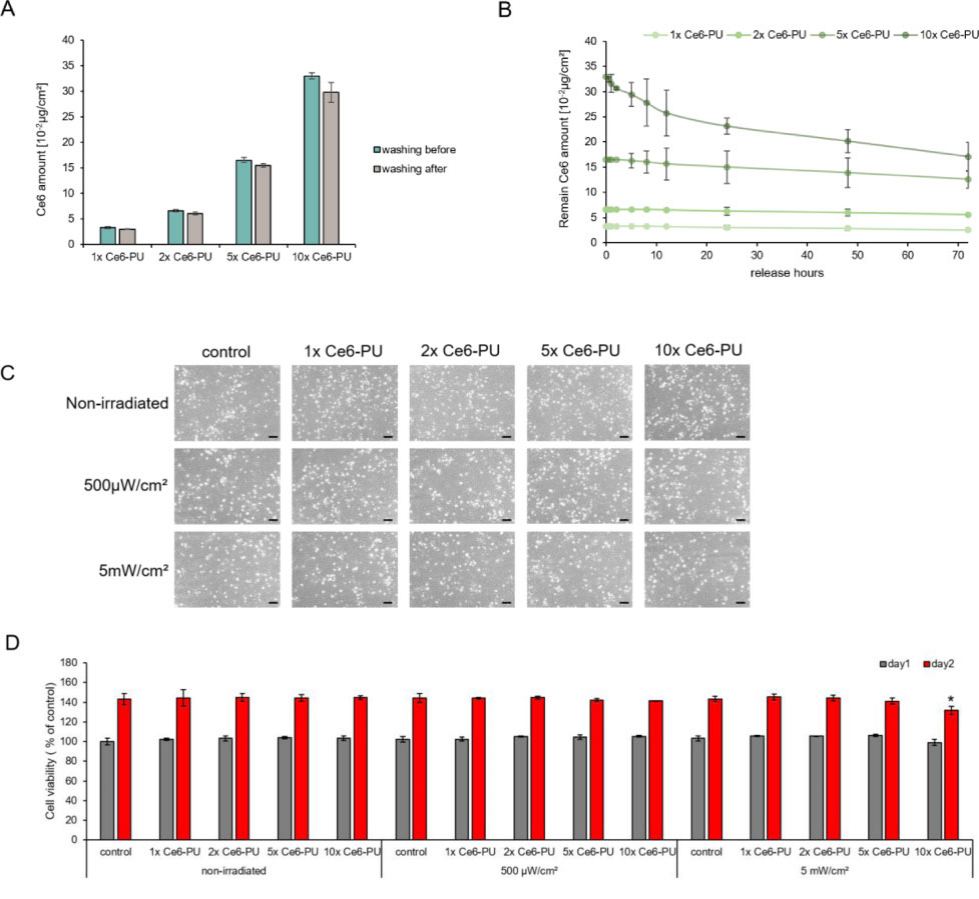
Confirmation of the *in vitro* Ce6 efflux concentration. In each experiment, DMSO was added to collect Ce6. The standard Ce6 diluted concentration was estimated by measuring the fluorescence of the samples. The concentration of Ce6 was calculated using the Ce6 standard curve. (**A**) The Ce6 leaking amount during the washing procedure. At each step of the washing procedure, the washing solution was collected and analyzed by fluorescence. Data are expressed as the mean ± SD. (**B**) The amount of Ce6 remaining during PBS incubation. The 1×, 2×, 5× and 10× Ce6-PU film was incubated in PBS and at specific time intervals. PBS was collected and the amount of Ce6 was confirmed by measuring the fluorescence. (**C**) Viability of cells in the solution released from the Ce6-PU film. Microscopic images of cell morphology at 48 h after irradiation. Scale bar = 100 µm (**D**) Quantification of the number of viable cells by MTT assay for 24 and 48 h. Data are expressed as the mean ± SD, *P < 0.05 vs. control.

Four Ce6 concentrations of Ce6-PU film were soaked in 70% EtOH for 30 min, and then washed five times with DW under the same conditions for Ce6-PET film sterilization. First, we gathered the sterilization solution in each step and then added DMSO to collect Ce6 to determine the Ce6 amount through the sterilization procedure. At each procedure, Ce6 was collected by adding DMSO to EtOH and DW. The PU film was used as a Ce6 delivery material. However, the PU film has porous structure properties. Thus, Ce6 included in PU could easily efflux. Some Ce6 was released through the sterilization procedure. Ce6 was washed out at different rates for each film. In case of the 1× Ce6-PU film, 8.6% of Ce6 was washed out compared with the amount when it was first fabricated. Interestingly, 7.8% of 2× Ce6-PU, 6.2% of 5× Ce6-PU and 9.7% of 10× Ce6-PU were washed out compared with the amount when it was fabricated (Fig. 4A).

To confirm the amount of photosensitizer released from the PU film, the Ce6-PU film was immersed in PBS for 30 min, 1, 2, 5, 8, 12, 24, 48 and 72 h after the sterilization step. The release of Ce6-PU from PBS was monitored by fluorescence in the time interval. For Ce6-PU, the maximum plateau was reached after 24 h. The slopes and plateaus showed a parallel trend. At 10× Ce6-PU, the film had a 10% released out of burst effect for 24 h and a slow-releasing Ce6 effect during a period of 72 h. The Ce6 amount was fully released after 72 h. After immersion in PBS for 72 h, 1× Ce6-PU film released 10%, 2× Ce6-PU film released 6% and 5× Ce6-PU film released 8% compared with the first loaded amount (Fig. 4B). Therefore, when Ce6-PU was added to the PU film and fabricated, a maximum of 20% of Ce6 was leaked out throughout the sterilization and release procedure.

Ce6 release is inefficient because it works in less amount than what you put in. Free Ce6 causes unintended effects under unintended light treatment. To determine the free Ce6 effects on cells, free Ce6 released from Ce6-PU film to EGM-2 was collected in order to verify the effect of cell viability. The cells were treated with the released media of Ce6 for 2 h. We compared the cell viability between high and low power densities of light. Under the low density of light (500 µW/cm^2^), cell viability was not affected by the interaction of free photosensitizer and light irradiation compared with cell viability in the control. However, under the high density of light (5 mW/cm^2^), Ce6 released from 10 × Ce6-PU affected HUVEC viability 2 days after LED irradiation (Fig. 4C and D).

### ROS generation comparison between the Ce6-PET and Ce6-PU films

**Figure 5.**
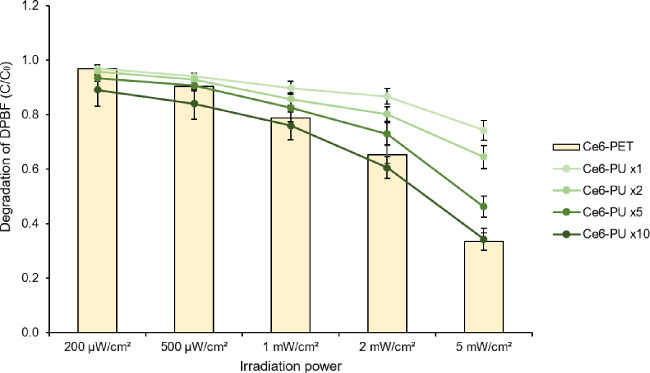
ROS Production comparison between Ce6-PET and Ce6-PU. The ratio between initial OD. and decomposition OD is shown as irradiation power. DPBF decomposition of the Ce6-PET film was represented as a bar graph, and that of the Ce6-PU film was represented as a line graph. Both films were irradiated at 200, 500 µW, 1, 2 mW and 5 mW/cm^2^ at 660 nm for 30 min. Data are expressed as the mean ± SD.

Different concentrations of Ce6-PU film were irradiated under the same conditions for 30 min to compare the effectiveness of ROS generation. Both Ce6-PET and Ce6-PU films were prepared ready to applicate on cells, thus both were washed in 70% EtOH for 30 min and rinsed five times with DW. Ce6-PET and Ce6-PU films were compared after same sterilization and washing procedure. After film preparation, each film was soaked in DPBF solution and irradiated with different power levels of red light. With the same light power level, Ce6-PET film triggered more DPBF degradation than Ce6-PU film, which contained the same amount of Ce6 as the Ce6-PET film when it was fabricated. The required amount of Ce6 varied according to the intensity of the LED power in order to produce a similar amount. A power level of 200 µW/cm^2^ requires a 2× Ce6-PU film to generate a similar level of ROS compared with the Ce6-PET film. A 500 µW/cm^2^ power requires a 5× Ce6-PU film compared with the Ce6-PET film. A power level of 1, 2 and 5 mW/cm^2^ requires a 10× Ce6-PU film compared with the Ce6-PET film (Fig. 5).

## Discussion

Dopamine is a useful surface coating material for biomedical applications. With regard to the application of dopamine on the surface of materials, various interactions, such as covalent bonds and coordination bonds, are involved in various ways. Since dopamine functional groups use covalent bonding to immobilize biomolecules, dopamine coated on the film is useful for surface modification and application in cell culture [52]. To increase the amount of immobilized photosensitizer, it is important to increase the chance of the functional group of dopamine to react. Dopamine concentration is an essential tool for controlling the roughness of surfaces. Dopamine at low concentrations functionalizes nanostructures by decreasing polydopamine particle formation efficiently. Low concentrations of dopamine self-polymerizes and causes interparticle aggregation that reduces polydopamine particle formation. However, the thickness of polydopamine is not constant at high concentrations of dopamine [53]. For this reason, it is important to determine the appropriate concentration of dopamine. Dopamine is easily oxidized to polydopamine by polymerization. Polydopamine adheres to all types of surfaces because of the presence of catechol moieties assisted by amino groups. Polydopamine has basic (NH_2_) and acidic (catechol-OH) sites, which can act as functional groups for further application [52]. Polydopamine has a unique chemical structure containing many functional groups that can be used to immobilize molecules covalently. Because of these characteristics, polydopamine has been used extensively for modulating cellular and tissue responses to materials [54]. In this study, a film was devised to generate extracellular ROS under LED irradiation through the excitation of photosensitizers. Thus, the efficiency of the film increases as the number of immobilized photosensitizers increases.

The degradation of DPBF is usually used to measure the extracellular ROS yield, which indicates that oxidation of DPBF is the most commonly used method to estimate the ROS concentration in media. The absorbance of DPBF decreased upon interaction with ROS and increased with increasing LED and irradiation power. The control group showed no degradation under light irradiation, since there was no ROS generation to interact with the DPBF. The DPBF decomposition study indicates that ROS are efficiently generated from photo-excited Ce6 molecules immobilized on the surface of the PET film. We confirmed that the higher the power of red-light irradiation on Ce6-PET film, the more DPBF was decomposed. Consequently, the results showed that ROS generation by Ce6-PET film depended on the power of the LED source.

ROS can influence cellular proliferation and differentiation by regulating the associated factors. Primarily, ROS can act as an activator in transcription factors and modulate cell signaling directly or indirectly. In particular, the transcription factors c-JUN and ATF are potential targets that are involved in ROS-mediated signaling. When JUN and p38 MPAK signaling is elevated, they contribute to their increased proliferative capacity [55]. ROS are also involved in the NF-κB signaling pathway, PI3K-Akt signaling pathway, and MAPKs signaling pathway to stimulate downstream targets and promote cellular proliferation [56, 57]. These cascades change the cell cycle and accelerate the S phase [36]. The transition from G1 to the S phase in the cell cycle is critical for the enhancement of cellular proliferation. Therefore, ROS could trigger the cell cycle signaling transition [58]. Extracellular ROS can increase growth factor production, such as vascular endothelial growth factor [59]. Growth factors bind to their receptors, and intracellular ROS is induced by signaling pathways [60]. Thus, extracellular ROS mediates intracellular ROS formation, and intracellular ROS contributes to the regulation of endothelial cell cycles. In our previous study [36], we found a relationship between extracellular ROS and cellular proliferation enhancement. In the experiment, ROS was generated by hematoporphyrin (Hp)-PU film. The Hp-PU film was irradiated at 510 nm, which corresponds to the maximum absorption of Hp in the visible light region. HUVEC growth was enhanced through ROS generated from Hp-PU after green light irradiation. Although different film fabrication methods and different photosensitizers were used in this study, we also demonstrated that extracellular ROS generated from the excited photosensitizer stimulates HUVEC growth.

There are many methods to immobilize photosensitizers on polymer films. Photosensitizers are immobilized by adsorption, entrapment, cross-linking and covalent bonding. Photosensitizers can be absorbed through Van der Waal’s forces, electrostatic interactions and hydrophobic interactions. In the entrapment method, a photosensitizer is not directly attached to the surface but entrapped within a polymeric network. A cross-linking method uses covalent bonding to create an irreversible linkage [61]. Although the designed Ce6-PU film, including a low amount of Ce6, released Ce6 could affect cell viability under a high density of light irradiation. Thus, these results demonstrated that Ce6-incorporated PU films could enrich ROS under light irradiation. In our previous study [36], the Hp-PU film was used after the photosensitizer was fully released from the film to avoid side effects. Here, we used Ce6-PU film as a comparison target to compare Ce6-PET on ROS generation. Therefore, Ce6-PU film was used after EtOH sterilization and washing with DW. Although direct comparison is impossible because of the different amounts of photosensitizer used and incorporated, the Ce6-PU film released a greater amount of photosensitizer than the Hp-PU film and generated a larger amount of extracellular ROS compared with the amount of photosensitizer. During PDT, photosensitizers have side effects associated with the low specificity of photosensitizers to target regions. PU film has excellent properties for use in experiments [62]. They have excellent elongation and tensile strengths, elastomeric memory, chemical resistance and low-temperature flexibility. However, swelling in the PU film reduces its hardness and diminishes the mechanical properties [63]. There are some problems when a photosensitizer is introduced to the film. Because of these problems, it was not easy to control the amount of Ce6 on the PU film in the experiment. When Ce6 is released from the film, free Ce6 can be released by irradiation with red light, and ROS can be created at an unintended place [64–66]. This unpredictable ROS production could affect cell viability. Thus, free Ce6 may lead to side effects due to systemic distribution and unintended remnants *in vivo*. In our study, we wanted to compare two different type of materials which include photosensitizer. Comparing PET and PU as materials, sterilization and washing procedure was carried out equally.

Therefore, the fabrication of PET films immobilized with photosensitizers by covalent bonds is a straightforward method to manage the generation of ROS using a small amount of photosensitizer compared with the design of Ce6-PU film by solvent casting. Through the Ce6-PET film technique, it is feasible to control ROS generation precisely by regulating the LED source power or irradiation time.

## Conclusion

In conclusion, we fabricated a Ce6-immobilized PET film via covalent bonding; this film produces controllable levels of extracellular ROS under red-light irradiation. In endothelial cells, ROS produced from the Ce6-PET film could effectively promote cell growth following red-light irradiation at an intensity of 500 μW/cm^2^ for 30 min. Thus, using the Ce6-PET film could generate a low level of ROS and stimulate HUVEC proliferation. In this study, we also demonstrated that Ce6 immobilized with covalent bonds on the PET film surface could produce ROS more efficiently than a Ce6-incorporated PU film synthesized by solvent casting. Therefore, we confirmed the possibility of surface modification using a photosensitizer based on chemical bonding through this approach. 

## Funding

This work was supported by a National Research Foundation of Korea (NRF) grant funded by the Korean government (MSIT; Nos 2017M3A9B3063638 and 2019R1A2C2005256). 

*Conflict of interest statement*. None declared. 
